# How to responsibly acknowledge research work in the era of big data and biobanks: ethical aspects of the Bioresource Research Impact Factor (BRIF)

**DOI:** 10.1007/s12687-017-0332-6

**Published:** 2017-09-25

**Authors:** Heidi Carmen Howard, Deborah Mascalzoni, Laurence Mabile, Gry Houeland, Emmanuelle Rial-Sebbag, Anne Cambon-Thomsen

**Affiliations:** 1 Centre for Research Ethics and Bioethics, Uppsala University, Uppsala, Sweden; 2 UMR 1027, Inserm, Université Toulouse III—Paul Sabatier, Toulouse, France; 3 Institute for Biomedicine, Eurac Research, Affiliated Institute of the University of Lübeck, Bolzano, Italy; 4 Plateforme Sociétale Genotoul, 37 allées Jules Guesde, Toulouse, France

**Keywords:** Biobank, Ethics, Recognition, Bioresource sharing, Bioresource Research Impact Factor, BRIF

## Abstract

Currently, a great deal of biomedical research in fields such as epidemiology, clinical trials and genetics is reliant on vast amounts of biological and phenotypic information collected and assembled in biobanks. While many resources are being invested to ensure that comprehensive and well-organised biobanks are able to provide increased access to, and sharing of biomedical samples and information, many barriers and challenges remain to such responsible and extensive sharing. Germane to the discussion herein is the barrier to collecting and sharing bioresources related to the lack of proper recognition of researchers and clinicians who developed the bioresource. Indeed, the efforts and resources invested to set up and sustain a bioresource can be enormous and such work should be easily traced and properly recognised. However, there is currently no such system that systematically and accurately traces and attributes recognition to those doing this work or the bioresource institution itself. As a beginning of a solution to the “recognition problem”, the Bioresource Research Impact Factor/Framework (BRIF) initiative was proposed almost a decade and a half ago and is currently under further development. With the ultimate aim of increasing awareness and understanding of the BRIF, in this article, we contribute the following: (1) a review of the objectives and functions of the BRIF including the description of two tools that will help in the deployment of the BRIF, the CoBRA (Citation of BioResources in journal Articles) guideline, and the *Open Journal of Bioresources* (OJB); (2) the results of a small empirical study on stakeholder awareness of the BRIF and (3) a brief analysis of the ethical dimensions of the BRIF which allow it to be a positive contribution to responsible biobanking.

## Introduction

Currently, a great deal of biomedical research in fields such as epidemiology, clinical trials and genetics is reliant on vast amounts of biological and phenotypic information collected and assembled in biobanks (Capocasa et al. 2016; Mabile et al. 2013). Maximising the potential of biobanks can greatly help us to better understand and manage health and disease (Altshuler et al. 2010; Harris et al. 2012). Biobanks, however, are relatively new research tools and vary enormously in many ways; they vary in size, scope and focus; samples can be collected from the general population, patients who have undergone a medical procedure or from deceased persons (Baker 2012). The data associated with and generated from these biospecimens (e.g. blood, saliva and other biospecimens) often require long-term storage and international sharing as well as advanced bioinformatics tools to maximise current and future research uses (Bravo et al. 2013; van Ommen et al. 2015). Although they are diverse in their aims and contexts, biobanks have been developed not only to offer storage and management of biospecimens and data, but they are meant to also provide responsible access to biospecimens and data as well as to support and facilitate sharing with different researchers.

While focus and resources are being placed to ensure that comprehensive and well-organised biobanks are able to provide increased access to and sharing of biomedical information (Harris et al. 2012), many barriers and challenges remain to such responsible and extensive sharing (Colledge et al. 2014). Problems related to how to adequately allow for sample and data sharing while protecting the privacy of individual research participants are still being widely discussed (Vaught et al. 2014). Another technical and often financial obstacle is that each biobank needs to keep up with the latest developments of analytical procedures. Germane to the discussion herein, an additional obstacle to collecting and sharing bioresources has been identified as the lack of proper recognition of researchers and clinicians who developed the bioresource (Colledge et al. 2013). Indeed, the efforts and resources invested in a bioresource can be enormous and such work should be easily traced and properly recognised.

As a beginning of a solution to the “recognition” problem, the Bioresource Research Impact Factor/Framework (BRIF) initiative was proposed almost a decade and a half ago and work on how to make the BRIF initiative into concrete and useful tools is currently ongoing. The concept of the BRIF was first proposed in 2003 (Cambon-Thomsen 2003) and has since been further developed (Bravo et al. 2015; Mabile et al. 2013; Mabile et al. 2016). As described by Mabile et al., the central aim of the BRIF is to construct a “quantitative tool to evaluate the impact of the use of a bioresource in research” (Mabile et al. 2013). In effect, the BRIF would allow for proper tracking of bioresource use which would also indirectly allow for the adequate recognition of persons involved in creating and sustaining such resources.

With the ultimate aim of raising awareness and understanding of the BRIF, this article provides: firstly, a review of the objectives and functions of the BRIF including the description of two tools that will help in the deployment of the BRIF, the CoBRA (Citation of BioResources in journal Articles) guideline, and the *Open Journal of Bioresources* (OJB). This review helps the reader understand the BRIF concept and the few tools already developed. Secondly, we present the results of a small empirical study on stakeholder awareness of the BRIF, which offers empirical data regarding what stakeholders in biobanking know of this novel framework and if they deem it useful. Finally, we offer a brief analysis of the ethical dimensions of the BRIF, which go beyond those of recognition, to allow readers to consider the potentially wide breadth of positive impacts the BRIF could have on to biobanking.

## Objectives and functions of the BRIF

**Fig. 1:**
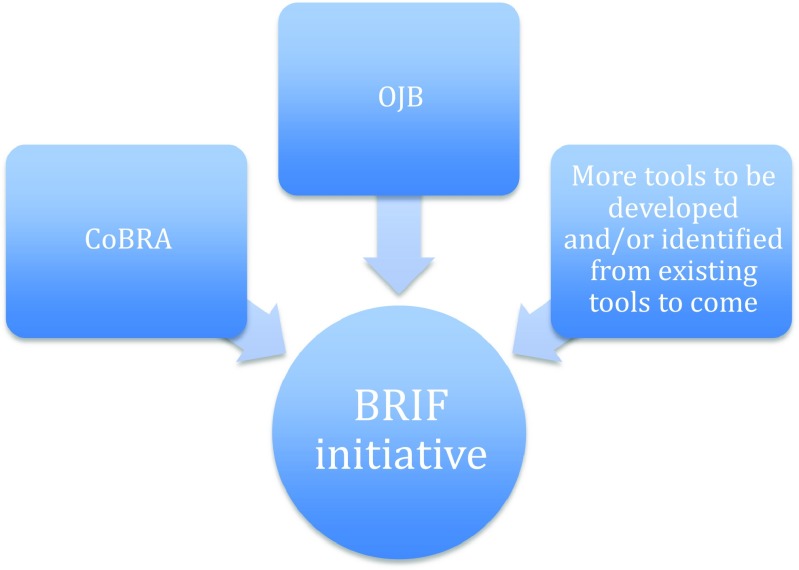
Summary of the tools that contribute to the BRIF initiative. Different tools (in boxes) have been developed to help in the development of the BRIF initiative towards a functional system of recognition of bioresources. The BRIF initiative is still in development and the existing tools include the following: (1) the CoBRA guidelines which give instructions on how to cite bioresources, http://www.equator-network.org/wp-content/uploads/2015/03/Cobra-check-list.pdf and (2) the *Open Journal of Bioresources* which publishes the description of different bioresources so that this publication can then be cited. Additional tools and resources will be identified and/or developed to help make the BRIF initiative funcational (e.g. DOI numbers, DataCite)

In practice, the BRIF is an ongoing initiative aiming to provide guidance and methodology for optimising recognition of bioresources, their use and their sharing. As a first step, the BRIF works to develop a framework that facilitates the accurate acknowledgement of bioresources through tracking the use of said resources in research. The impact of these resources will then eventually be measured by relevant fair metrics and performance indicators (De Castro et al. 2013; Hofman et al. 2013; Mabile et al. 2016). The basis of the BRIF concept is to make it possible to systematically trace the use of a bioresource in academic literature and to quantify its use by relevant metrics. The tacit goal is to ensure responsible acknowledgement of work as well as to encourage the actors involved in bioresource work to share these resources efficiently (Mabile et al. 2016). The implementation of the BRIF thus depends on its ability to meet stakeholders’ requirements and to incorporate it into existing systems of practice and parameters (Mabile et al. 2013). “Tracing the use of a bioresource is the first step in this process and new tools have been or are being developed to make it feasible: the CoBRA guideline (Citation of BioResources in journal Articles), the Open Journal of Bioresources (OJB) and BRIF metrics” (Mabile et al. 2016). Ideally, a unique digital identifier assigned through existing mechanisms (e.g. DOI) (Kauffman and Cambon-Thomsen 2008) should be used to cite and acknowledge the use of bioresources in publications and funding grants. It could then be followed and captured in a quantitative tool to measure the impact of bioresource use in research (Cambon-Thomsen et al. 2011). (Fig. 1).

### Tools that have been developed to help fulfil the aims of the BRIF initiative

1. The CoBRA (Citation of BioResources in journal Articles) guideline

Already, the CoBRA guideline (Bravo et al. 2015, http://www.equator-network.org/wp-content/uploads/2015/03/Cobra-check-list.pdf) has been developed by a dedicated BRIF working group to standardise citation of bioresources in scientific articles (Napolitani et al. 2016). The CoBRA recommends that each individual bioresource used in research should be mentioned in the “Methods section” of articles and should be cited as an individual reference (Mabile et al. 2016). The CoBRA citation scheme will permit the traceability of bioresource use in the literature, and it will also permit the traceability of secondary use of bioresources (Mabile et al. 2016). “One way to enforce CoBRA use in articles is to include it in instructions to reviewers and editorial assistants as part of the checklist used to process manuscripts” (Mabile et al. 2016). Another way is to explain how to cite the bioresource via CoBRA in the material transfer agreement (MTA) or the data transfer agreement (DTA) (Bravo et al. 2015; Napolitani et al. 2016) required by biobanks.

2. The *Open Journal of Bioresources* (OJB)

A second tool to help track bioresources comes in the form of a new journal called The *Open Journal of Bioresources* (OJB, http://openbioresources.metajnl.com) which “features peer-reviewed short papers helping researchers to locate and cite bioresources with high reuse potential”. The OJB is a so-called meta-journal with articles that are citable, and such citations can be tracked. The idea behind OJB is to provide a permanent “marker” article so that users can always cite a bioresource they have accessed or referred to. It is important to note that a bioresource article does not replace a research article (e.g. where original analyses of data are presented). It is meant to describe the resource as such, not to report any results obtained using the resource. Such results are to be reported in independent research articles that will, ideally, cite the descriptive marker article published in OJB. The latter describes the methods used to create the resource, how it is preserved/organised, its reuse potential and criteria for access. The articles are published in accordance with a structured template and they are peer reviewed for coherence, clarity, completeness and accuracy of the information provided. Such marker articles aim to increase the awareness of the biobank/bioresources and facilitate location of existing resources for potential users; they do not report their actual use or any results generated from their use (Mabile et al. 2016). It must be underlined that any publication describing a resource could be considered a marker article; but often, results are included in classical publications and authors are those who used the resource as well as those who set it up and maintain it. Using a marker article means that attribution of recognition for the latter is only given to those authors who actually performed that work, and as such is more transparent and coherent.

Admittedly, such tools could be used much more systematically than “reputation” for evaluating the activity and impact of a bioresource over time, as they facilitate traceability and provide a means to quantify the use of a bioresource. When taken into account in assessing “researchers/contributors” contribution to science, this would encourage facilitating and increasing the use of bioresources to maximise impact, hence fostering their sharing and their quality as maximising quality resources would increase reuse/sharing. A virtuous cycle would occur: the higher the quality, the more frequent will be the solicitations; the more one shares, the more one’s impact increases, the more one is inclined to share (Mabile et al. 2013). The idea is to facilitate a measurable recognition, not only for the scientist proposing the projects or doing analyses, but for those who work to ensure the quality of bioresource, to get ethical approval, to maintain the resource and to organise its sharing in practice. But, it is also meant to empower all the participants, the data subjects in the resource who could follow more easily what is being done with a resource they are included in (even in case of anonymisation, they can still identify the resource, although not their individual sample) (Kauffman and Cambon-Thomsen 2008). It increases transparency and it also makes it feasible for the institution funding the bioresource to accurately be informed of the use of the resources they fund. So all stakeholders, including editors, should find an interest in such tools and framework (Mabile et al. 2013).

## Awareness of the BRIF: what does the empirical data show?

While the BRIF may be a good idea in theory, for it to work in practice, stakeholders must be aware of the initiative and learn to use the aforementioned tools in their work. However, based on a questionnaire distributed to stakeholders in genetics at two meetings in the Spring of 2013, we found that awareness of the BRIF initiative was very low among these stakeholders.

More specifically, a seven-item questionnaire was elaborated in order to assess the level of awareness of the BRIF by researchers and clinicians in genetics. A short paragraph describing the BRIF initiative was also included on the reverse side of the questionnaire (hence, this acted partly as an educational or awareness-raising endeavour). The first question asked if the respondent was aware of the BRIF before being asked to fill in the questionnaire, and if so, how he/she found out about it. The second question asked if the stakeholder thought that a tool like the BRIF would be useful and why. The five remaining questions were meant to assess the context of the responder (what kind of research-related work he/she performed and the type(s) of biobanks or registries with which (s)he worked). The anonymous hardcopy (printed on paper) questionnaire was distributed at two different genetics meetings: EuroGentest meeting (Prague, March 2013) and European Society of Human Genetics—ESHG (Paris, June 2013).

A total of 88 questionnaires were returned from stakeholders in genetics; 60 questionnaires were returned from the EuroGentest meeting and 28 from the ESHG meeting. A small majority (48%) of respondents had research as their main professional activity while 41% reported working mainly in the clinic, 7% had administrative jobs and 4% chose “other”. Respondents worked in 18 different European countries, as well as in Russia, Canada, China, Qatar and Saudi Arabia. Sixty percent of respondents worked in some capacity with biobanks, registries or repositories. The large majority of respondents (89%, 78/88) were not aware of the BRIF before being asked to fill out the questionnaire. The remaining 11% were made aware of the BRIF mostly through direct contact with researchers involved in the initiative, or colleagues discussing the initiative, as well as at meetings where the BRIF was presented. Despite the low previous awareness of the BRIF, a majority of 63% of respondents thought a tool like the BRIF would be useful, 35% had no opinion and 2% said they did not think it would be useful (without elaborating further). Of those who elaborated on this question, the following are the types of remarks left by respondents:

**Table:** 

“I think it can make the research ‘easier’, ‘faster’”
“To enhance experts’ willingness to contribute to databases”
“Because of the important work for such infrastructure may be better acknowledged”
“Good to have an appropriate measurement system”
“It could help in the collaboration between researchers & biobanks”
“It increases awareness of available resources”
“It will provide a common stage to reference”
“It would create good incentives for biobank use”
It would “avoid identical data in multiple study merged analysis”

It thus appears that there is a positive willingness to use such an approach from the community of geneticists. This echoes the experience of authors (ACT, LM) who have presented the BRIF for various audiences and found that it was received very positively. However, the means to disseminate information about the BRIF initiative seem to have been inadequate, at least until 2013. This is partly due to the difficulty to get publications in major journals about an ongoing initiative. This is a challenge to the progression of the BRIF initiative, since it is precisely during its development (rather than after its completion) that community and stakeholder input is needed. Furthermore, for the BRIF to be a successful framework, it must be used by a majority of stakeholders in biobanking.

Taking the results of this study into account, dissemination activities have been set up more actively since 2013: several articles have appeared in journals addressing different communities; the BRIF initiative is presented at conferences and meetings worldwide, mainly in the biobanking community as well as in the editorial and IST world; input from scientific societies and consortia (e.g. the European Society of Human Genetics, European Middle Eastern and African Society for Biopreservation and Biobanking, International Society for Biological and Environmental Repositories, Public Population Project in Genomics (P3G), BioSHaRE project) has been received; the BRIF was included in the BBMRI-ERIC infrastructure (http://www.bbmri-eric.eu) 2015 work programme and editors’ workshops were organised in 2013 and 2015.

Furthermore, as a way to incentivise the use of the CoBRA guideline, it is referred to by different networks and organisations; for example, it is mentioned by the EQUATOR Network (the key network for biomedical guidelines, http://www.equator-network.org/reporting-guidelines/), and by BBMRI-ERIC, which encourages that it be mentioned in material and data transfer agreements. Ongoing contacts and discussions with journal editors also serve to push its inclusion in the instructions to authors and reviewers. Additional work is currently being conducted with the Research Data Alliance (which has an aim to facilitate all types of data sharing, https://www.rd-alliance.org) as an interest group to target at the institutional level. Through these different activities, it is likely that there is greater awareness of the BRIF now than there was in 2013 (including greater awareness of the CoBRA guideline and the *Open Journal of Bioresources*). We consider that it would take a number of years before awareness would increase significantly. We, therefore, assess that a survey of stakeholders in the coming couple of years would be informative and a good moment to reassess the need for additional dissemination activities.

## Ethical aspects of the BRIF

Indeed, the BRIF may be a good idea in theory and may also bring with it many practical reasons for implementing it. For example, the BRIF offers the possibility to fairly recognise and acknowledge the work needed to develop and maintain bioresources as well as the work needed to promote their use. The consequent use of the BRIF as a quantitative evaluator for grant applications by individual and institutional applicants, as well as its ability to allow a more effective measure of the use of the resource is an additional good reason to want to implement it. Less evident, but equally important, are the ethical and social implications (and by extension, potential policy implications) that the implementation of the BRIF could allow. In our opinion, the BRIF could have a large role to play in the recognition, enhancement, and ongoing support of salient values in biobanking research; in particular, we highlight herein the following three values:

- Transparency in the use of the bioresource(s) (allowing for a type of accountability metrics for the institutions)
- Fairness in the recognition of the contribution made by institutions and individuals (which can be valuable for grants and career advancement)
- Informing and potentially empowering patients and research participants by allowing clear feedback to them regarding the use of the provided materials and data.

### The BRIF allows for transparency and monitoring of the bioresources’ activities which can help promote the responsible and effective use of bioresources

Biobank activities have long been under scrutiny, especially, with regard to the ethical implications of biobanking practices around storage, primary use, secondary use and sharing of biomaterials. Concerns include how (securely and efficiently) materials are collected, shared (or not), with which institutions and for what purpose(s).

To help make measures of transparency meaningful and upheld (in practice) by biobank stakeholders, the BRIF, which can be considered as a type of accountability framework, can help reveal how, in reality, a biobank policy is (or is not) being followed. However, tracking projects and publications using a given collection of materials, if done in a manual way (auditing the institutes and the biobanks and analysing all the MTA and cooperation agreements etc.), would require large amounts of (human) resources. This, among other reasons, is why it is uncommon to provide a trace of the uses of bioresources. If assessing the list of projects that used certain bioresources was only a click away, this could allow for the possibility of monitoring the use of the bio-samples and could have a constructive impact on different, albeit overlapping aspects:

- Accounting for the effective use of bioresource: The bioresource is not only a “passive” collection but provides a useful service for the scientific community, which can access bio-samples and perform research on the collections.
- Serve as an indicator for measuring cost/efficiency: The tracking system could also allow for access to details needed to answer the questions regarding the cost/benefit of a bioresource. For example, how much has the bioresource been used? Did the uses lead to results?
- Indirectly indicate the perceived quality of the bioresource by the scientific community: The fact that a bioresource is more used than others can be an indirect indicator of perceived or actual quality. In order to access a bioresource, researchers need to be confident that biomaterials and data have been collected and stored according to adequate standards (CoE 2006; OECD 2006; Parodi 2015) and according to scientifically sound procedures.
- Promote fairness in the application of access procedures to bioresources: The claim that bioresources should be available for advancing science based on fair assessment and access policies relies on the good faith of individuals towards the work done by an institutional review board. It is difficult, however, to obtain data to show compliance with policies on the effect of access policies on the distribution of biomaterials. The integration of access policies and the use of third party access committees to regulate the distribution and the use of bioresources and data (Mascalzoni et al. 2015; Shabani et al. 2016) reflects the attempt to overcome potential conflicts of interest between the institution that collected data (that still retains the interest to exploit the potential of a resource for which big investments were made) and the aim of maximising the exploitation of the resource for the sake of science. Fairness in access constitutes an important goal. It is commonly known that easier access may be granted to “friend scientists” or to former collaborators, or conversely, denied access may occur to rival scientists. The BRIF could help provide an overview of the access provided to different bioresources, thus potentially showing if certain institutions have been benefiting from more (or less) access per request made. The BRIF may also facilitate finding answers to the following questions: does the use of samples comply with the original stated scope and aims of the collection? And, which institutions are using the bioresources?

By providing an overview of the use of the bioresource in practice, the BRIF can provide an incentive for the responsible application of access and sharing policies. It can also help in identifying flaws and provide data that could subsequently be used for calibrating and adjusting access policies.

More specifically, for several of the above points, the use of marker articles (described earlier) which clarify the conditions of use, the sharing policies and any conditions for the usage of bioresources may be an adequate transparent and citable way of promoting fair access rules and, confronted with the actual use measured by the BRIF, it may provide a way to assess the gap, if any, between the use and the intended policies.

### The BRIF can increase fairness in publication policies by recognising more accurately the different works done by different actors

The trend for fair and increased (e.g. open) access to bioresources (especially for data) has been heavily promoted by funding agencies and by Biomedical journals. This said, there remains nonetheless resistance by various stakeholders to granting (greater) access to bioresources. Among the reasons for this resistance is the lack of measures to grant credit to institutions and ensure fair and accurate recognition of the work performed by groups that planned, studied, collected and prepared the collections and the datasets. A recent editorial by Taichman et al. (2016) regarding a proposal from the International Committee of Medical Journal Editors (ICMJE) emphasises the need for alternative incentives to recognise contribution in the context of clinical trial data. However, much of the issues they raise can also apply to biobanking. “…those who generate and then share clinical trial data sets deserve substantial credit for their efforts. Those using data collected by others should seek collaboration with those who collected the data. However, because collaboration will not always be possible, practical, or desired, an alternative means of providing appropriate credit needs to be developed and recognised in the academic community” (Taichman et al. 2016).

For a long time, co-authorship has been recognised as the main reward mechanism and has been regarded as far more important than the promotion of sharing. In fact, it has often been an accepted precondition for sharing. In recent years, however, this type of “reward authorship” has been heavily criticised for not being compliant with authorship guidelines (ICMJE 2016) which require substantial contribution on different levels (planning, execution of experiments, writing/revising article) to the completion of the work and drafting of the paper itself.

Creating and sustaining bioresources requires substantial investments, which may lead to a legitimate institutional interest in verifying appropriate use of them and seeing this investment recognised. Creating a bioresource also involves scientific work in designing and planning of the resource characteristics. Indeed, the effective dissemination of bio-samples and research results should be associated with mechanisms for acknowledging intellectual contribution and originality through rules of authorship or other means for recognising substantial contribution (Mascalzoni et al. 2015). The BRIF can contribute along with MTA/DTA (which will still define, where appropriate, authorship conditions) to the recognition of the contributions made by institutions (Mascalzoni et al. 2015). However, such recognition is also the precondition for promoting greater access and sharing of samples and data. Implication of this includes the following:

- Accurately recognising the contribution of institutions and different contributors to the bioresource. By linking the BRIF to the “published bioresource description” (i.e. the marker article) (Bravo et al. 2015), virtually every type of contribution can be acknowledged from logistical organisation to scientific designing of the collection. In the long run, this will count and have an impact on one’s CV, thus recognising work done on different levels to create and maintain a bioresource and not only an honour “authorship” for the head of the bioresource.
- The BRIF can be effective in the promotion of a bioresource by displaying the use of data and samples by other institutions, which already evaluated the collection as scientifically valuable to be used and whose use also lead to published results. This could, in turn, create a virtuous cycle by generating more requests for the bioresource.

### The BRIF can help in making information available for patients, research participants and stakeholders in general about the use of bioresources

The possible concrete impact of the BRIF for patients and stakeholders was originally anticipated as a potential goal as described by Kauffman and Cambon-Thomsen (2008) (Kauffman and Cambon-Thomsen 2008): “This would empower research participants with a tangible key to follow the use of the collection in which their sample and data belong, even in case of future anonymisation”. Currently, it is virtually impossible for patients and research participants to follow the use of their data and samples over time. At the time of consent, it is impossible to foresee all the possible future uses of the biomaterials and data which, therefore, means that patients and research participants are often only informed in a general way about the possible uses and the policies to ensure security and fair use. While the use of ongoing information strategies such as those involved in dynamic consent (Budin-Ljosne et al. 2017; Kaye et al. 2015) can help with this problem to some extent, this remains an open-ended dilemma in informed consent literature. Furthermore, it is very difficult to account for all the uses of a single bioresource because many different projects are run at the same time, by different institutions and very often, results are not returned to the bioresource directly. While, in theory, there is agreement on the need for more transparent policies and the possibility to track uses and projects and this has been advocated by streams of the ELSI scholarship that ask for more respect for individual/community choices (Gainotti et al. 2016; Kaye et al. 2015), it is, in practice, very difficult to ensure traceability and account for different uses. The BRIF could then be utilised as a way for patients and stakeholders to follow the developments and the effective use of the bioresource. The BRIF could easily allow patients to have information on the institution website without much investment. In order to allow participants to trace the dynamic use of the resource to which they contributed, one could foresee that the unique identifier given to a resource could be provided to participants and, in addition to following the BRIF reported on the website of the biobank involved, they could themselves search (using the bioresource ID) for more precise information if they so wish. The BRIF framework could then:

- Ensure traceability: The BRIF, once implemented, can account for published results of projects completed using different bio-collections and by doing so, ensure traceability of the actual different uses of sets of samples, data and results from the bioresource. This can constitute a primary source for patients and participants in order to be able to follow results and publications even if the original institution recruiting them has no resources to convey such information to them. As much as finding technical papers may be problematic for a layperson, this is still better than no information at all and better than filtered results.
- Showcase conformity within the approved use of data: By allowing participants to look at who accessed biomaterials and for what purpose the BRIF (via the CoBRA guideline for published results or via the unique identifier of the collection for any other case) could be used to assess compliance with informed consent purposes, limits for sharing and approved uses by ethical boards.
- Allow for participant empowerment by allowing them some form of oversight: Patients/research participants may, through the BRIF, follow the use of the bioresource, comment on it, monitor it and ask for more effective use, or diverse uses if necessary.
- Increase trust by transparent use: Being aware of what kind of research the bioresource is contributing to (including the research area/theme and being able to trace who are the researchers and from which groups/universities) might lead to increased trust by patients and stakeholders.

## Conclusion

While the BRIF is still a project in the making, and the awareness of its existence by relevant stakeholders is rather low, positive support for its development and the clear positive impacts it could have for biobanks and biobank stakeholders are promising. Ongoing efforts are being made to actively increase awareness of the BRIF and to obtain valuable stakeholder input throughout different stages of its development. Indeed, for awareness and use of the BRIF to become widespread, there is a need to gain recognition and endorsement of the developed tools (e.g. CoBRA, OJB) by different levels of stakeholders. These different stakeholders include the following: (i) institutional research stakeholders such as universities, national institutes, and research infrastructures which could provide incentives and/or nudges for their researchers to use the tools; (ii) scientific organisations such as professional societies or consortia which could provide guidance to their members about using the tools and (iii) educational and editorial stakeholders who could include using the tools in their good practice guidelines for graduate students and in the instructions for authors and reviewers, respectively.

If widely implemented, the BRIF could have an important positive impact on the responsible use of bioresources and related datasets. Clearly, we have focussed herein on the positive aspects of the BRIF initiative, as we believe that these clearly outweigh the potential negative aspects. This said, there could also be negative points to consider. As with any method or metric that attempts to measure use (or citations), such as the journal impact factor, perfection is impossible. There is always a possibility that the measuring method may not be adequate for all resources, and that it will not be used or interpreted properly within each resources’ specific context. (Ramsden 2009) Undeniably, the term “impact factor” carries a lot of connotations, and the word framework has been added to the BRIF term over the years (from Bioresource Research Impact Factor to Bioresource Research Impact Factor/Framework) to move somewhat away from the strict notion of the impact factor and to more adequately reflect the work-in-progress and the overall approach which includes many different tools.

With respect to the ethical aspects and impacts of the BRIF, by providing an easily accessible overview of existing uses of a bioresource, the BRIF can promote a better use of the bioresource through a virtual cycle and promote the virtues of transparency, fair recognition of work, as well as allow for better informing and potential empowering research participants and patients. One could consider the last two decades as a period of time where a lot of resources were spent creating and developing biobanks and datasets. Now that many different bioresources exist and function, we can consider the time ripe to prioritise the use of funds, time and (human) resources to ensure that these bioresources are used in a responsible and sustainable way; the BRIF clearly contributes to this aim, and as such should be further supported both financially and politically.
